# Cerebrospinal Fluid Immune Cell Alterations in Women With Neuropsychiatric Long COVID

**DOI:** 10.1093/infdis/jiaf468

**Published:** 2025-09-08

**Authors:** Benjamin Orlinick, Sameet Mehta, Lindsay McAlpine, Saba Khoshbakht, Sofia Fertuzinhos, Allison Nelson, Jennifer Chiarella, Bibhuprasad Das, Vansh Patel, Paraskevas Filippidis, Michael J Corley, Serena S Spudich, Shelli F Farhadian

**Affiliations:** Section of Infectious Diseases, Yale School of Medicine, New Haven, Connecticut, USA; Section of Infectious Diseases, Yale School of Medicine, New Haven, Connecticut, USA; Division of Neurological Infections, Yale School of Medicine, New Haven, Connecticut, USA; Department of Cellular and Molecular Medicine, KOC University, Istanbul, Turkey; Center for Bioinformatics, Cushing Medical Library, Yale School of Medicine, New Haven, Connecticut, USA; Division of Neurological Infections, Yale School of Medicine, New Haven, Connecticut, USA; Division of Neurological Infections, Yale School of Medicine, New Haven, Connecticut, USA; Section of Infectious Diseases, Yale School of Medicine, New Haven, Connecticut, USA; Section of Infectious Diseases, Yale School of Medicine, New Haven, Connecticut, USA; Department of Pathology, Yale School of Medicine, New Haven, Connecticut, USA; Department of Medicine, University of California San Diego, San Diego, California, USA; Division of Neurological Infections, Yale School of Medicine, New Haven, Connecticut, USA; Center for Brain and Mind Health, Yale School of Medicine, New Haven, Connecticut, USA; Section of Infectious Diseases, Yale School of Medicine, New Haven, Connecticut, USA; Division of Neurological Infections, Yale School of Medicine, New Haven, Connecticut, USA; Department of Epidemiology of Microbial Diseases, Yale School of Public Health, New Haven, Connecticut, USA

**Keywords:** long COVID, neuropsychiatric symptoms, cerebrospinal fluid, RNA sequencing, post-viral syndrome

## Abstract

**Background:**

Women are disproportionately affected by neuropsychiatric symptoms following recovery from acute COVID-19. However, whether there are central nervous system-specific changes in gene expression in women with neuropsychiatric Long COVID (NP-Long COVID) remains unknown.

**Methods:**

Twenty-two women with and 10 women without NP-Long COVID were enrolled from New Haven, Connecticut, and the surrounding region and consented to a blood draw and large volume lumbar puncture. Total RNA was extracted from cerebrospinal fluid (CSF) cells and peripheral blood mononuclear cells (PBMC). Polyadenylated RNA was sequenced, and differential expression analyses were performed.

**Results:**

Both CSF and PBMC samples showed differential gene expression associated with Long COVID status. There were CSF-specific differentially expressed genes in people with Long COVID, including in genes related to oxidative stress, reactive oxygen species, and P53 response, indicating compartment-specific immune responses. Some pathways were dysregulated in both the CSF and PBMC of Long COVID compared with controls, including those related to androgen response, MTORC1 signaling, and lipid metabolism.

**Conclusions:**

Women with NP-long COVID show compartment-specific, transcriptional profiles in the CSF with evidence of enrichment in cellular stress pathways. These results underscore the importance of examining CSF-specific molecular profiles to better understand post-viral neurological syndromes.

## Teaser

Women with neuropsychiatric Long COVID exhibit unique cerebrospinal fluid transcriptional profiles, revealing central nervous system-specific immune, metabolic, and stress pathway alterations not observed in peripheral blood, highlighting compartmentalized molecular mechanisms underlying persistent post-infectious neurological symptoms.

The coronavirus disease 2019 (COVID-19) pandemic, caused by the severe acute respiratory syndrome coronavirus 2 (SARS-CoV-2), has had unprecedented global health implications beyond its acute respiratory manifestations. Some patients report a constellation of symptoms long after recovering from acute infection, collectively termed Long COVID [1]. The World Health Organization (WHO) defines Long COVID as symptoms that continue after acute infection or new symptoms that occur after 3 months from the onset of probable or confirmed SARS-CoV-2 infection, persist for at least 2 months, and cannot be explained by an alternative diagnosis [2]. The etiology of long COVID symptoms remains unknown.

Neuropsychiatric manifestations represent a particularly challenging aspect of Long COVID, with mounting evidence suggesting a significant and disproportionate impact on women [3–5]. Neuropsychiatric Long COVID (NP-Long COVID) symptoms include cognitive difficulties, commonly known as brain fog, alterations in mood, and persistent fatigue [6]. While estimates of the prevalence of neurological and psychiatric symptoms in people with Long COVID vary across studies due to differences in study populations, definitions of Long COVID, and methods of assessment, collectively, they underscore a potentially substantial impact of Long COVID on neurological and mental health. Yet the underlying molecular mechanisms driving these neuropsychiatric sequelae remain poorly understood.

Our study aims to provide a detailed molecular characterization of NP-Long COVID in the central nervous system (CNS) by conducting a comparative analysis of gene expression profiles in peripheral blood mononuclear cells (PBMC) and cerebrospinal fluid (CSF) in people with self-reported Long COVID compared to those without. Our findings suggest a complex molecular landscape in the CSF underlying neuropsychiatric symptoms following COVID-19 infection in women.

## METHODS

### Participants and Enrollment

**Figure 1.**
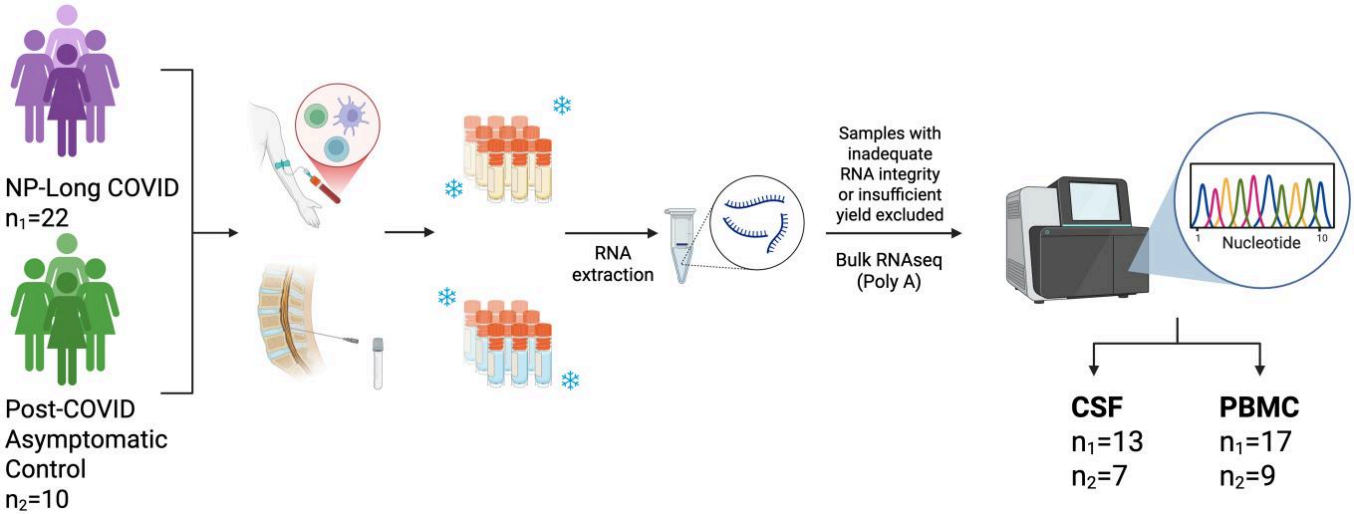
Study overview. Women with self-reported neuropsychiatric long COVID (NP-long COVID) and post-COVID asymptotic controls enrolled and underwent blood draw and large volume lumbar puncture at the same visit. Total CSF cell pellet and PBMC were collected, and RNA was extracted for bulk RNA sequencing.

Women with a documented history of COVID-19 and with self-reported Long COVID with neurological and/or psychiatric symptoms were enrolled from New Haven, Connecticut, and surrounding areas to the COVID Mind Study at Yale University (Figure 1). Classification of Long COVID followed the World Health Organization's formal definition and was supported by standardized surveys and comprehensive medical and psychiatric history taking. Participants also underwent clinician review of medical records and reported symptoms to enhance phenotypic characterization. At their study visit, participants consented to a blood draw and lumbar puncture as well as surveys on medical and social histories, demographics, and a comprehensive neuropsychological testing battery. Individuals were excluded from enrollment if they were known to be pregnant, or had a major pre-existing neurological disorder (eg, seizure, multiple sclerosis). Menopausal and contraceptive status were determined through self-report using standardized intake surveys administered during the study visit. This included questions on menstrual history, current use of hormonal contraceptives, and menopause status. Women with a history of documented SARS-CoV-2 infection without persistent symptoms were enrolled as controls.

This study was approved by the Yale University Institutional Review Board. All participants provided written informed consent.

### Sample Processing and Storage

Participants provided between 70 and 100 cc of blood and between 15 and 30 cc of CSF during the study visit. Fresh CSF was centrifuged at 300 *× g* for 10 minutes and the supernatant was aspirated until only 500 µL remained. The cell pellet and leftover supernatant were then transferred to a microcentrifuge tube, spun at 300 × *g* for 10 minutes, and the supernatant was aspirated until only 50 µL remained. Peripheral blood mononuclear cell were isolated using a Ficoll gradient and SepMate tubes. An average of 5–10 million viable PBMCs were cryopreserved per vial, based on post-isolation trypan blue staining. Cerebrospinal fluid cell pellets were stored at −80°C in either RNALater or 90% fetal bovine serum with10% Dimethyl sulfoxide (DMSO). Peripheral blood mononuclear cells were cryopreserved in 90% Human heat-inactivated serum and 10% DMSO in liquid nitrogen.

### Nucleic Acid Extraction

DNA and RNA were extracted from thawed CSF cells using the AllPrep DNA/RNA Micro Kit (CAT No 80284). The DNA/RNA was extracted from thawed PBMC using the AllPrep DNA/RNA/miRNA Universal Kit (CAT No: 80224).

### Library Preparation and Sequencing Protocols

PolyA sequencing was performed on total RNA from both CSF and PBMC samples at the Yale Center for Genome Analysis. CSF sequencing libraries were prepared using the NEBNext Single Cell/Low Input RNA Library Prep Kit for Illumina (E6420L), and samples that achieved an RNA integrity number (RIN) of ≥ 7.0, had visible ribosomal peaks, and 500 pg of input material were sequenced using 100 bp paired-end sequencing on the Illumina NovaSeq × (cat No). If RIN was < 7.0 but there was sufficient starting material and visible ribosomal peaks, sequencing was also attempted. Peripheral blood mononuclear cells sample libraries were prepared using the Kapa mRNA Hyper Prep Kit, and samples that achieved an RNA quality number (RQN) ≥ 7.0 and an RNA concentration of ≥ 50 ng/µL were sequenced using 100 bp paired-end sequencing on the Illumina NovaSeq X. In total, 20 CSF samples and 26 PBMC samples had RNA of sufficient quality (RNA samples meeting a minimum RIN of ≥7.0 [or RQN ≥7.0 for PBMCs], presence of visible ribosomal peaks on electropherograms, and sufficient input concentration as per library prep kit guidelines) to proceed with sequencing.

### Differential Gene Expression and Clustering Analyses

Differential Expression analysis was performed in R on raw counts from PBMCs and CSF separately using the package DESeq2 [7]. Results were then extracted and differentially expressed genes (DEGs) were identified using an adjusted *P* value < .05. Variance stabilizing transformation was applied to the normalized DESeq2 dataset to facilitate principal component analysis visualization. Heatmaps applying hierarchical clustering on samples and DEGs were generated using the R package pheatmap using normalized counts [8]. Normalized counts were also used to generate volcano plots using EnhancedVolcano [9].

### Gene Set Enrichment Analysis

Gene Set Enrichment Analysis was performed on normalized PBMC and CSF counts in GSEA v4.3.3. Both analyses were run with 1000 permutations based on phenotype, gene symbols collapsed, and randomization mode set to equalize and balance. Genes were ranked based on Signal2Noise and enrichment was based on the Human Molecular Signatures Data Base (MSigDB) Hallmark gene sets v2024 [10–12]. Over-representation analysis was performed on overlapping DEGs across tissues using g:profiler [13].

### Immune Deconvolution

TPM values (normalized gene expression estimates from bulk transcriptome analysis) were used to computationally estimate cell type frequencies through the QuanTIseq algorithm, which is designed for human immune cell deconvolution [14, 15]. Because the samples were processed in aggregate, statistical differences were determined by a Wilcoxon ranked-sum test, adjusting for multiple comparisons with Benjamini–Hochberg correction.

## RESULTS

### Study Cohort

The demographics of the study cohort are summarized in *Table 1*. Participants with NP-Long COVID and asymptomatic post-COVID controls (henceforth “controls”) were well matched by age (median age 48.5 vs 41.5; *P* = .35), race (81.82% white vs 90% white; *P* > .999), and comorbidities, including hypertension and Type II diabetes. There were no significant differences in history of psychiatric illness or current antidepressant use. However, control participants were more likely to report a history of smoking and a history of substance use disorder. Among participants, 43.75% of women enrolled in the study were postmenopausal, and 25% reported hormonal contraceptive use at the time of the study visit. The hormonal status of women in the 2 groups did not differ. Participants underwent blood draw and lumbar puncture (LP) a median of 365 days after acute COVID-19 infection (range: 100–1157 days in NP-Long COVID and 72–1042 days in controls).

**Table 1.** Clinical and Demographic Features of Study Participants

	NP-Long COVID (n = 22)	Control (n = 10)	*P* Value
Age, median (range)	48.50 (23–63)	41.50 (19–73)	0.35
CSF data available	13	7	
PBMC data available	17	9	
Fully vaccinated, n (%)	19 (86.36%)	10 (100)	0.5343
Days since acute infection, median (range)	373 (100–1157)	137 (72–1042)	0.5747
Hospitalized during acute COVID-19, n (%)	5 (22.73)	1 (10)	0.6367
Race/ethnicity, n (%)	…	…	
Black	3 (13.64)	0 (0)	0.5343
White	18 (81.82)	9 (90)	>0.999
`Other	1 (4.55)	1 (10)	0.5343
Hispanic	3 (13.64)	0 (0)	0.5343
Drug and alcohol use, n (%)	…	…	
Current/former smoker	6 (27.27)	7 (70)	*0.0494
Current smoker	1 (4.55)	1 (10)	0.5343
Former smoker	5 (22.73)	6 (60)	0.0557
Never smoker	17 (77.27)	4 (40)	0.0557
History of alcohol use disorder	1 (4.55)	2 (20)	0.2238
History of substance use disorder	0 (0)	3 (30)	*0.0220
Metabolic comorbidities	…	…	
Hypertension, n (%)	7 (31.82)	3 (30)	>0.999
Type II diabetes, n (%)	2 (9.09)	1 (10)	>0.999
BMI, median (range)	27.69 (19.13–48.71)	31.57 (21.95–49.63)	0.3265
Reproductive health, n (%)	…	…	
Postmenopausal	11 (50)	3 (30)	0.4461
Premenopausal on hormonal birth control	5 (22.73)	3 (30)	0.6808
Premenopausal not on hormonal birth control	6 (27.27)	4 (40)	0.6828
Mental health, n (%)	…	…	
History of psychiatric illness (diagnosis or self-report)	9 (40.91)	6 (60)	0.4501
Current antidepressant use	9 (40.91)	7 (70)	0.2524

### Neuropsychiatric Long COVID has a Distinct Transcriptional Profile in Both Cerebrospinal Fluid and Blood When Compared with Controls

**Figure 2.**
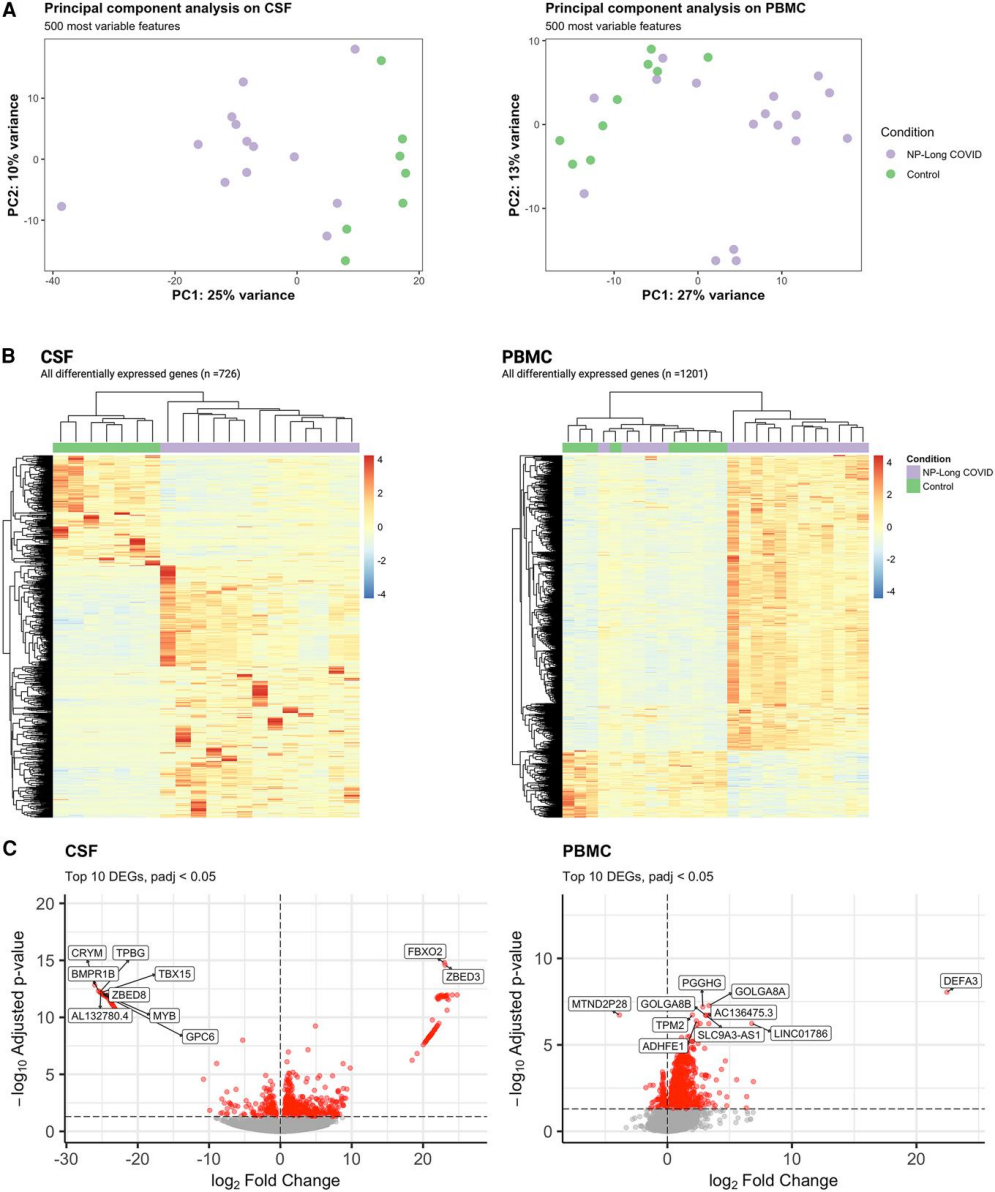
Neuropsychiatric Long COVID associates with distinct transcriptional profiles in the PBMC and CSF compared with controls, and these profiles are compartment-specific. *A*, Principal component analysis of CSF and PBMC demonstrates that CSF samples separated by long COVID status along PC1. *B*, Heatmaps of DEGs (*p*_adj_ < .05) in CSF and PBMC. *C*, Volcano plots demonstrating the top (highest −log *p*_adj_) DEGs in CSF and PBMC.

Both CSF and PBMCs demonstrate strikingly different transcriptional profiles based on NP-Long COVID status. Using principal component analysis, CSF samples separated clearly based on NP-Long COVID status along PC1. In contrast, in PBMC, NP-Long COVID status did not drive separation along PC1 or PC2 (Figure 2*A*). Similarly, using hierarchical clustering with DEGs, CSF samples clustered by NP-Long COVID status; in contrast, PBMC samples did not cluster by NP-Long COVID status (Figure 2*B*). Differentially expressed genes in the CSF showed markedly higher changes in expression levels between disease states when compared with DEGs in the blood (Figure 2*C*). In PBMC, relative to the CSF, there were few genes with a large magnitude of difference in gene expression between NP-Long COVID and controls. Cerebrospinal fluid cells contained 85 DEGs with a log_2_ Fold Change > |20| compared with only 1 in PBMC.

### Divergent Expression Profiles of Differentially Expressed Genes in the Cerebrospinal Fluid Compared with Blood of NP-Long COVID

**Figure 3.**
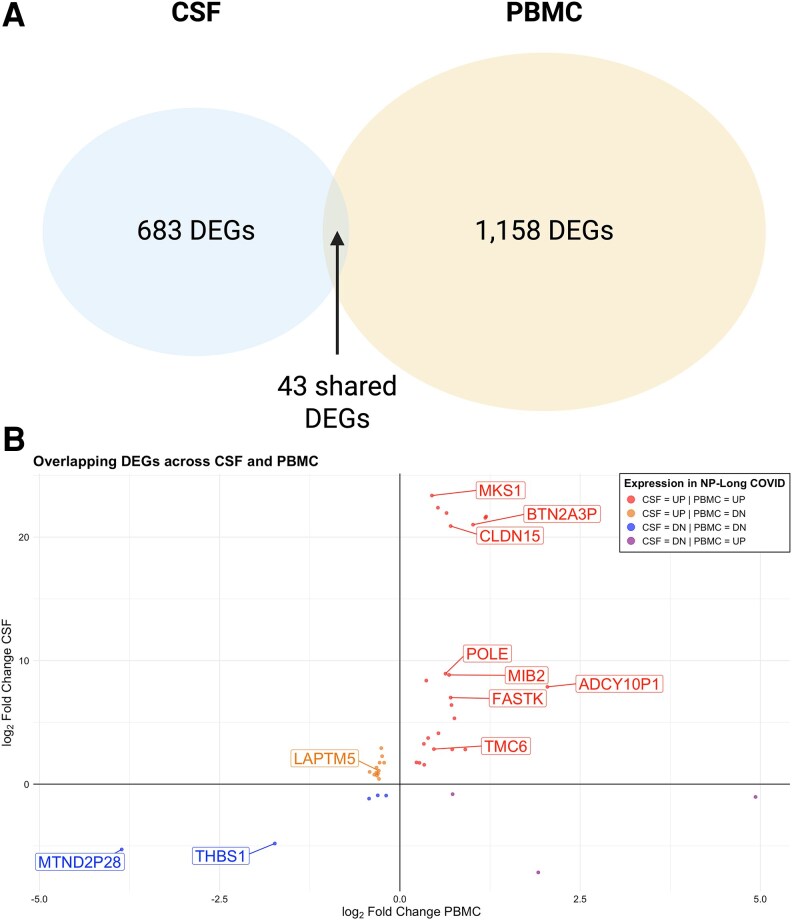
Little overlap in DEGs between CSF and PBMC. Differentially expressed genes sharing the same HGNC Gene Symbol were examined in each tissue. *A*, Of the 1927 DEGs in CSF and PBMC, only 43 are shared between compartments (*p*_adj_ < .05). *B*, All 43 shared DEGs are visualized in a scatterplot of log_2_Fold Change in CSF against log_2_Fold Change in PBMC, but only those that achieve *p*_adj_ < .01 in both tissues are labeled. Differentially expressed genes are divided into 4 categories concerning their expression in NP-Long COVID: upregulated in both PBMC and CSF (red, n = 23), upregulated in CSF and downregulated in PBMC (orange, n = 12), downregulated in both PBMC and CSF (blue, n = 5), and downregulated in CSF and upregulated in PBMC (purple, n = 3).

Among genes that were differentially expressed in NP-Long COVID compared with controls, there was scant overlap between DEGs in the 2 compartments (Figure 3). Of the 1884 unique DEGs in CSF and PBMC, only 43 genes were differentially expressed in both CSF and PBMC (Figure 3*A*). Furthermore, only 28 of these genes showed consistent directionality across compartments (ie, shared direction of change in NP-Long COVID vs controls) (Figure 3*B*). Using g:profiler to perform over-representation analysis, we examined the genes that were differentially expressed in both CSF and blood, but in different directions in the CSF versus the blood, when comparing NP-Long COVID to controls. These diverging genes demonstrated over-representation in immune-related pathways, including TNFR1-induced NF-kB signaling (genes: *RACK1* and *UBC*) and innate immune response to SARS-CoV-1 (genes: *PPIA* and *UBC*). Among the genes that were significantly overexpressed in the CSF of NP-Long COVID while being underexpressed in the Blood of NP-Long COVID was *LAPTM5,* which encodes a lysosomal transmembrane protein crucial for brain function and has been implicated in neurological diseases and immune-related processes.

### Distinct Genetic Pathways Define the Cerebrospinal Fluid Versus Blood Transcriptional State in Neuropsychiatric Long COVID

**Figure 4.**
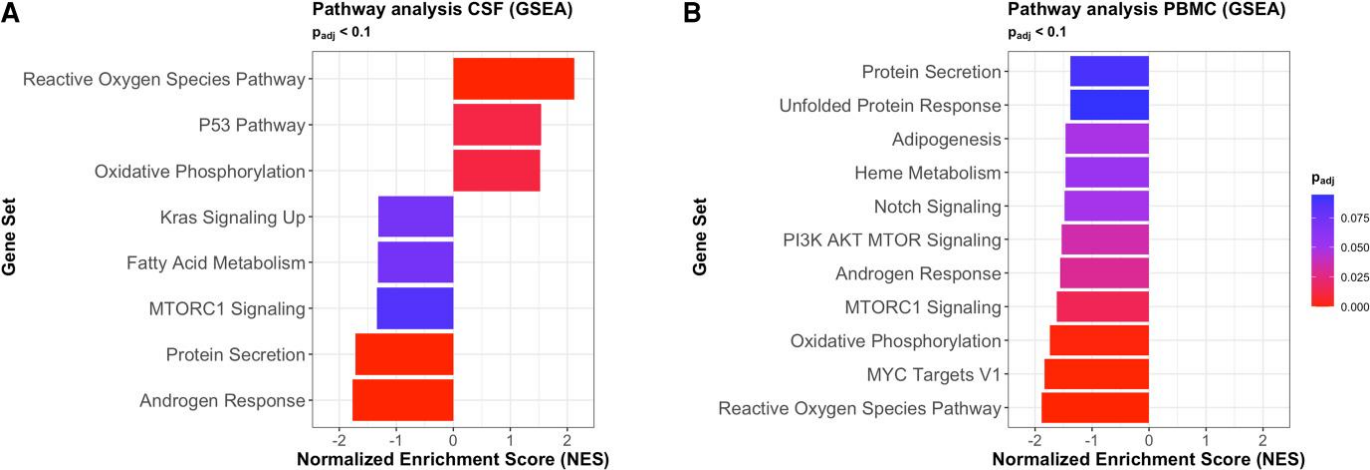
Gene Set Enrichment Analysis using the Human Molecular Signatures Database Hallmark gene sets was performed on CSF and PBMC and reveals distinct enrichment patterns in each compartment, suggesting a compartment-specific response to NP-Long COVID. *A*, Pathways that were differentially enriched in the CSF based on disease status included reactive oxygen species, P53, and oxidative phosphorylation (enriched in CSF of women with NP-Long COVID) and androgen response, protein secretion, MTORC1 signaling, fatty acid metabolism, and KRAS signaling (enriched in the CSF of controls). *B*, No pathways are enriched in PBMC of women with NP-Long COVID, and pathways enriched in controls are unique relative to CSF except MTORC1 signaling and androgen response.

We next identified genetic pathways that were enriched in the disease-associated gene sets by applying gene set enrichment analysis to the DEGs in the CSF and PBMC (Figure 4). We observed that androgen response, mechanistic Target Of Rapamycin complex (MTORC)-related pathways, fatty acid related pathways, and protein secretion were enriched in controls (ie, downregulated in women with NP-Long COVID) in both PBMCs and CSF. In contrast, while reactive oxygen species and oxidative phosphorylation were both significantly enriched in PBMCs and CSF, their directionalities differ between tissues. In CSF, reactive oxygen species and oxidative phosphorylation are enriched in women with NP-Long COVID (Figure 4*A*). In PBMC, however, reactive oxygen species and oxidative phosphorylation were enriched in asymptomatic women and downregulated in women with NP-Long COVID (Figure 4*B*)

Several genetic pathways displayed differential expression in either CSF or PBMC of NP-Long COVID, but not both. The Kirsten rat sarcoma virus (KRAS) signaling was impaired in CSF only, and MYC targets V1, notch signaling, heme metabolism, and unfolded protein response pathways were impaired in PBMCs but not CSF. Furthermore, reactive oxygen species, the P53 pathway, and oxidative phosphorylation were all enriched in the CSF of women with NP-Long COVID, but no hallmark pathways appeared to be enriched in the PBMC of women with NP-Long COVID.

### Cell Frequencies do not Differ by Neuropsychiatric Long COVID Status in the Cerebrospinal Fluid or Peripheral Blood Mononuclear Cells After Adjusting for Multiple Comparisons

Cell type proportions were estimated using the QuanTiSeq cell deconvolution tool for RNA-seq data. After adjusting for multiple comparisons, there were no statistical differences in any cell type proportions in either CSF or blood when comparing NP-Long COVID to controls (Supplementary Figure 1). However, CSF from women with NP-Long COVID displayed a trend for lower levels of M2 Macrophages compared with the CSF of controls.

## DISCUSSION

Cerebrospinal fluid is a “window to the brain” that can be accessed in living people and which can shed light on neuroinflammatory and neurodegenerative conditions. By comparing the CSF to blood transcriptome, we identify tissue-specific immune dysregulation in the CNS in women with NP-Long COVID. Relative to PBMC, CSF samples demonstrate significantly more genes with large (>20-fold) changes in expression level in the disease state. Furthermore, pathways involved in cellular stress response, including reactive oxygen species, oxidative phosphorylation, and P53 signaling, are all enriched in the CSF of women with NP-Long COVID but not in the PBMC.

In CSF, the top upregulated gene in NP-Long COVID was *FBXO2 (log2FC 22.99; p*_adj_  *= 1.58 × 10^−15^)*. The *FBXO2* plays a critical role in waste degradation and lysophagy in the CNS [16]. The most downregulated gene in the CSF of NP-Long COVID was *CRYM,* which encodes crystallin mu (log_2_ Fold Change = −26.02; *p*_adj_ = 1.47 × 10^–13^). This crystallin, negatively regulated by T3 thyroid hormone, functions as a ketimine reductase in the brain and binds nicotinamide adenine dinucleotide (NADH) or NADPH to metabolize imine bonds, and plays an important role in amino acid metabolism, particularly lysine [17, 18]. Lysine is a precursor of the neurotransmitter glutamate and can exert neuroprotective effects in mammalian brains through M2 macrophage polarization [19, 20]. Notably, loss of *CRYM* in mouse striatal astrocytes dysregulates synaptic function. These results suggest that NP-Long COVID is associated with metabolic and neuroimmune alterations in the CSF compartment, including disruption of lysosomal clearance pathways and amino acid metabolism.

Interestingly, while oxidative phosphorylation appears significantly downregulated in the PBMC, we observe the opposite trend in CSF. Oxidative phosphorylation upregulation in CSF may reflect a heightened metabolic demand or mitochondrial stress in CNS-resident immune cells, potentially in response to chronic neuroinflammation or tissue injury in NP-Long COVID. Conversely, the downregulation of oxidative metabolism in PBMCs could suggest systemic immune exhaustion or a shift toward glycolytic metabolism commonly observed in chronic inflammatory states. These findings underscore the compartmentalized nature of NP-Long COVID and highlight the importance of evaluating CNS and systemic immune responses in parallel.

While men typically experience more severe acute COVID-19 [21], studies show women are at higher risk for Long COVID [22, 23].. However, It is not yet clear why there is an increased prevalence of NP-Long COVID in women compared with men. Emerging evidence suggests that hormonal, immunological, and genetic factors may contribute to the observed differences in susceptibility and manifestations of long COVID symptoms between men and women [24–26]. However, comprehensive molecular studies exploring the intricate pathways responsible for these differences are limited, particularly in the CSF and CNS. Our finding of decreased androgen response in NP-Long COVID aligns with a growing body of evidence implicating androgens in COVID-19 pathophysiology that may underlie sex differences in susceptibility to NP-Long COVID. Notably, however, recent evidence suggests testosterone levels can significantly predict long COVID symptom burden irrespective of biological sex and may correlate with sex specific immune signatures of long COVID in men and women [4]. Future investigations include concurrent measurement of circulating sex steroids, which would allow for correlation with immune transcriptional changes.

Our results also demonstrated the downregulation of fatty acid metabolism pathways in CSF samples from people with NP-Long COVID. Fatty acids are essential for maintaining brain health, serving as both structural components of cell membranes and energy substrates for neural cells. Long-chain fatty acids, particularly omega-3 fatty acids such as docosahexaenoic acid, are essential components of brain endothelial cell membranes and are transported across the blood brain barrier (BBB) via specialized transporters like Mfsd2a [27]. Disruptions in fatty acid metabolism have been associated with various neurological conditions, including neurodegenerative diseases and neuroinflammatory disorders [28–30]. The reduced expression of fatty acid metabolism genes in NP-Long COVID CSF may therefore indicate compromised neural energy metabolism and potential myelin dysfunction.

We also closely examined gene expression changes in Long COVID that were unique to the CSF. Specifically, we found elevated expression of *LAMPTM5* in the CSF and decreased levels in the PBMC of NP-Long COVID. The LAPTM5 is a transmembrane receptor associated with lysosomes; it has been implicated in numerous disease processes, including cell death, immune response, autophagy, and inflammation [31]. The LAPTM5 is involved in toll-like receptor-mediated neuroinflammation in macrophages, microglial activation in response to neuronal apoptosis, downregulation of T cell receptors and T cell activation, and may have a protective role in cerebral ischemia/reperfusion injury [32–35].

Several limitations to the study must be acknowledged. The relatively small number of CSF samples limits the statistical power to detect subtle gene expression changes or rare cell populations; some transcriptional differences could have been missed due to limited power. While no statistically significant differences in cell type proportions were detected after correction for multiple testing, we cannot fully rule out subtle cell compositional effects. Single-cell resolution studies will be required to disentangle these effects more precisely. The classification of NP-Long COVID was based on self-reported symptoms, which introduces the potential for misclassification. The variability in time since acute infection among participants (ranging from approximately 3 months to over 3 years), may contribute to heterogeneity in immune transcriptional signatures. Our study did not include men, and thus we cannot be sure that similar findings do not underlie long COVID symptoms in men. Additionally, cryopreservation of biospecimens may have introduced artificial stress-related transcriptional changes, complicating the interpretation of some pathways. Future studies that incorporate fresh, non-cryopreserved CSF samples are therefore important to validate key findings. Moreover, future transcriptomic studies on NP-Long COVID should also adopt harmonized biospecimen handling protocols across centers to minimize batch-related effects and isolate true biological signal. In addition, while focusing on women allowed us to investigate hormonal and transcriptional mechanisms specific to this disproportionately affected group, our findings may not apply to men. Our results should be considered hypothesis-generating for future mixed-sex studies, which are essential to determine whether similar compartmentalized immune responses and androgen-related transcriptional changes occur in men with long COVID. The cross-sectional design of this study precludes assessment of how transcriptional signatures evolve over time in NP-Long COVID. While our findings capture a snapshot of gene expression many months post-infection, they do not reveal whether the observed changes are persistent, resolving, or progressive. Future longitudinal transcriptomics, ideally combined with neuroimaging, fluid biomarkers, and clinical outcomes, will be crucial for identifying biomarkers of persistence and potential targets for treatment.

Despite these caveats, using CSF transcriptomics, we uncovered distinct molecular alterations in the CNS of women experiencing NP-Long COVID, with the CSF exhibiting unique transcriptional changes not mirrored in peripheral blood. These included disruptions in immune regulation, metabolic activity, and cellular stress responses that may contribute to neurocognitive symptoms. The findings offer potential insight into why some women develop persistent neurological symptoms following SARS-CoV-2 infection. The observed compartment-specific dysregulation of oxidative stress, lipid metabolism, and androgen signaling pathways may inform therapeutic development. For instance, CNS-targeted antioxidants, neuroprotective lipids or agents modulating neuroendocrine signaling could be explored in future clinical trials. Moreover, the identification of CSF-enriched transcriptional signatures, such as elevated LAPTM5 expression or CRYM downregulation, may serve as candidate biomarkers for NP-Long COVID diagnosis or monitoring. These targets will need to be further validated in larger, longitudinal cohorts using orthogonal techniques, including protein quantification or imaging markers.

Overall, our results underscore the importance of examining compartment-specific molecular profiles to better understand postviral neurological syndromes. Future work should prioritize prospective, longitudinal studies with clinically adjudicated diagnoses, fresh sample collection, and deeper integration of hormonal and neuroimmune analyses to clarify the mechanisms underlying NP-Long COVID and better inform targeted therapeutic strategies.

## Supplementary Material

jiaf468_Supplementary_Data

## Supplementary Data

Supplementary materials are available at *The Journal of Infectious Diseases* online (http://jid.oxfordjournals.org/). Supplementary materials consist of data provided by the author that are published to benefit the reader. The posted materials are not copyedited. The contents of all supplementary data are the sole responsibility of the authors. Questions or messages regarding errors should be addressed to the author.

## References

[1] Su S, Zhao Y, Zeng N, et al Epidemiology, clinical presentation, pathophysiology, and management of long COVID: an update. Mol Psychiatry 2023; 28:4056–69. 37491461 10.1038/s41380-023-02171-3

[2] Post COVID-19 condition (Long COVID) . Available at: https://www.who.int/europe/news-room/fact-sheets/item/post-covid-19-condition.

[3] Sylvester SV, Rusu R, Chan B, et al Sex differences in sequelae from COVID-19 infection and in long COVID syndrome: a review. Curr Med Res Opin 2022; 38:1391–9. 35726132 10.1080/03007995.2022.2081454

[4] Silva J, Takahashi T, Wood J, et al Sex differences in symptomatology and immune profiles of long COVID. medRxiv 2024.02.29.24303568, Available at: 10.1101/2024.02.29.24303568, 2 March 2024, preprint: not peer reviewed.

[5] Gorenshtein A, Leibovitch L, Liba T, Stern S, Stern Y. Gender disparities in neurological symptoms of long COVID: a systematic review and meta-analysis. Neuroepidemiology 2024; 1:15. 10.1159/000540919 PMC12324708 39159607

[6] Graham EL, Clark JR, Orban ZS, et al Persistent neurologic symptoms and cognitive dysfunction in non-hospitalized COVID-19 ‘long haulers'. Ann Clin Transl Neurol 2021; 8:1073–85. 33755344 10.1002/acn3.51350 PMC8108421

[7] Love MI, Huber W, Anders S. Moderated estimation of fold change and dispersion for RNA-Seq data with DESeq2. Genome Biol 2014; 15:550. 25516281 10.1186/s13059-014-0550-8 PMC4302049

[8] Kolde R. pheatmap: Pretty Heatmaps . R package version 1.0.13. **2025**. Available at: https://github.com/raivokolde/pheatmap.

[9] Blighe K, Rana S, Lewis M. EnhancedVolcano: publication-ready volcano plots with enhanced colouring and labeling. doi:10.18129/B9.bioc.EnhancedVolcano, R package version 1.26.0. **2025**. https://bioconductor.org/packages/EnhancedVolcano.

[10] Subramanian A, Tamayo P, Mootha VK, et al Gene set enrichment analysis: a knowledge-based approach for interpreting genome-wide expression profiles. Proc Natl Acad Sci U S A. 2005; 102:15545–50. 16199517 10.1073/pnas.0506580102 PMC1239896

[11] Liberzon A, Birger C, Thorvaldsdóttir H, et al The molecular signatures database (MSigDB) hallmark gene set collection. Cell Syst 2015; 1:417–25. 26771021 10.1016/j.cels.2015.12.004 PMC4707969

[12] Mootha VK, Lindgren CM, Eriksson K-F, et al PGC-1α-responsive genes involved in oxidative phosphorylation are coordinately downregulated in human diabetes. Nat Genet 2003; 34:267–73. 12808457 10.1038/ng1180

[13] Kolberg L, Raudvere U, Kuzmin I, et al G:profiler—interoperable web service for functional enrichment analysis and gene identifier mapping (2023 update). Nucleic Acids Res 2023; 51:W207–12. 37144459 10.1093/nar/gkad347 PMC10320099

[14] Finotello F, Mayer C, Plattner C, et al Molecular and pharmacological modulators of the tumor immune contexture revealed by deconvolution of RNA-Seq data. Genome Med 2019; 11:34. 31126321 10.1186/s13073-019-0638-6 PMC6534875

[15] Sturm G, Finotello F, Petitprez F, et al Comprehensive evaluation of transcriptome-based cell-type quantification methods for immuno-oncology. Bioinformatics 2019; 35:i436–45. 31510660 10.1093/bioinformatics/btz363 PMC6612828

[16] Liu EA, Schultz ML, Mochida C, Chung C, Paulson HL, Lieberman AP. Fbxo2 mediates clearance of damaged lysosomes and modifies neurodegeneration in the Niemann-Pick C brain. JCI Insight 2020; 5:e136676. 32931479 10.1172/jci.insight.136676 PMC7605537

[17] Hallen A, Cooper AJL, Jamie JF, Haynes PA, Willows RD. Mammalian forebrain ketimine reductase identified as μ-crystallin; potential regulation by thyroid hormones. J Neurochem 2011; 118:379–87. 21332720 10.1111/j.1471-4159.2011.07220.x

[18] Hallen A, Cooper AJL, Smith JR, Jamie JF, Karuso P. Ketimine reductase/CRYM catalyzes reductive alkylamination of α-keto acids, confirming its function as an imine reductase. Amino Acids 2015; 47:2457–61. 26173510 10.1007/s00726-015-2044-8

[19] Papes F, Surpili MJ, Langone F, Trigo JR, Arruda P. The essential amino acid lysine acts as precursor of glutamate in the mammalian central nervous system. FEBS Lett 2001; 488:34–8. 11163791 10.1016/s0014-5793(00)02401-7

[20] Cheng J, Tang J-C, Pan M-X, et al l-lysine confers neuroprotection by suppressing inflammatory response *via* microRNA-575/PTEN signaling after mouse intracerebral hemorrhage injury. Exp Neurol 2020; 327:113214. 31987833 10.1016/j.expneurol.2020.113214

[21] Scully EP, Haverfield J, Ursin RL, Tannenbaum C, Klein SL. Considering how biological sex impacts immune responses and COVID-19 outcomes. Nat Rev Immunol 2020; 20:442–7. 32528136 10.1038/s41577-020-0348-8 PMC7288618

[22] Subramanian A, Nirantharakumar K, Hughes S, et al Symptoms and risk factors for long COVID in non-hospitalized adults. Nat Med 2022; 28:1706–14. 35879616 10.1038/s41591-022-01909-w PMC9388369

[23] Tsampasian V, Elghazaly H, Chattopadhyay R, et al Risk factors associated with post−COVID-19 condition: a systematic review and meta-analysis. JAMA Intern Med 2023; 183:566–80. 36951832 10.1001/jamainternmed.2023.0750 PMC10037203

[24] Gu J, Zhang J, Liu Q, Xu S. Neurological risks of COVID-19 in women: the complex immunology underpinning sex differences. Front Immunol 2023; 14:1281310. 38035090 10.3389/fimmu.2023.1281310 PMC10685449

[25] Klein SL, Flanagan KL. Sex differences in immune responses. Nat Rev Immunol 2016; 16:626–38. 27546235 10.1038/nri.2016.90

[26] Hosseinzadeh S, Afshari S, Molaei S, Rezaei N, Dadkhah M. The role of genetics and gender specific differences in neurodegenerative disorders: insights from molecular and immune landscape. J Neuroimmunol 2023; 384:578206. 37813041 10.1016/j.jneuroim.2023.578206

[27] Nguyen LN, Ma D, Shui G, et al Mfsd2a is a transporter for the essential omega-3 fatty acid docosahexaenoic acid. Nature 2014; 509:503–6. 24828044 10.1038/nature13241

[28] Tracey TJ, Steyn FJ, Wolvetang EJ, Ngo ST. Neuronal lipid metabolism: multiple pathways driving functional outcomes in health and disease. Front Mol Neurosci 2018; 11:10. 29410613 10.3389/fnmol.2018.00010 PMC5787076

[29] van der Velpen V, Teav T, Gallart-Ayala H, et al Systemic and central nervous system metabolic alterations in Alzheimer's disease. Alzheimer’s Res Ther 2019; 11:93. 31779690 10.1186/s13195-019-0551-7 PMC6883620

[30] Shamim A, Mahmood T, Ahsan F, Kumar A, Bagga P. Lipids: an insight into the neurodegenerative disorders. Clin Nutr Exp 2018; 20:1–19.

[31] Zhang M, Liang M-j, Zhang D-m, et al The function and mechanism of LAPTM5 in diseases. Biomed Pharmacother 2024; 178:117237. 39096616 10.1016/j.biopha.2024.117237

[32] Glowacka WK, Alberts P, Ouchida R, Wang J-Y, Rotin D. LAPTM5 protein is a positive regulator of proinflammatory signaling pathways in macrophages. J Biol Chem 2012; 287:27691–702. 22733818 10.1074/jbc.M112.355917 PMC3431655

[33] Origasa M, Tanaka S, Suzuki K, et al Activation of a novel microglial gene encoding a lysosomal membrane protein in response to neuronal apoptosis. Brain Res Mol Brain Res 2001; 88:1–13. 11295227 10.1016/s0169-328x(01)00005-5

[34] Ouchida R, Yamasaki S, Hikida M, et al A lysosomal protein negatively regulates surface T cell antigen receptor expression by promoting CD3zeta-chain degradation. Immunity 2008; 29:33–43. 18619870 10.1016/j.immuni.2008.04.024

[35] Zhang Z, Wang L, Wang Z, et al Lysosomal-associated transmembrane protein 5 deficiency exacerbates cerebral ischemia/reperfusion injury. Front Mol Neurosci 2022; 15:971361. 36046710 10.3389/fnmol.2022.971361 PMC9423384

