# Supplementary material for: Cerebrospinal Fluid Immune Cell Alterations in Women With Neuropsychiatric Long COVID
Source: J Infect Dis. 2025 Sep 8;233(1):e109–17. doi: 10.1093/infdis/jiaf468 (PMC12811860; doi:10.1093/infdis/jiaf468)
Supplement: jiaf468_Supplementary_Data [file jiaf468_supplementary_data.zip › Figure S1-legend.docx]

***Figure S1:* Immune cell subset differences in the CSF and PBMC of NP-long COVID.** Bulk Transcript per million (TPM) values were used to computationally estimate immune cell type frequencies using quanTIseq with the R package immunedeconv. After adjusting for multiple comparisons, there are no statistical differences in cell frequencies by long covid status in CSF or PBMC. However, uncorrected p-values suggest CSF from women with NP-long covid may have lower levels of M2 macrophages.
