# Supplementary figures and images for: Cerebrospinal Fluid Immune Cell Alterations in Women With Neuropsychiatric Long COVID
Source: J Infect Dis. 2025 Sep 8;233(1):e109–17. doi: 10.1093/infdis/jiaf468 (PMC12811860; doi:10.1093/infdis/jiaf468)

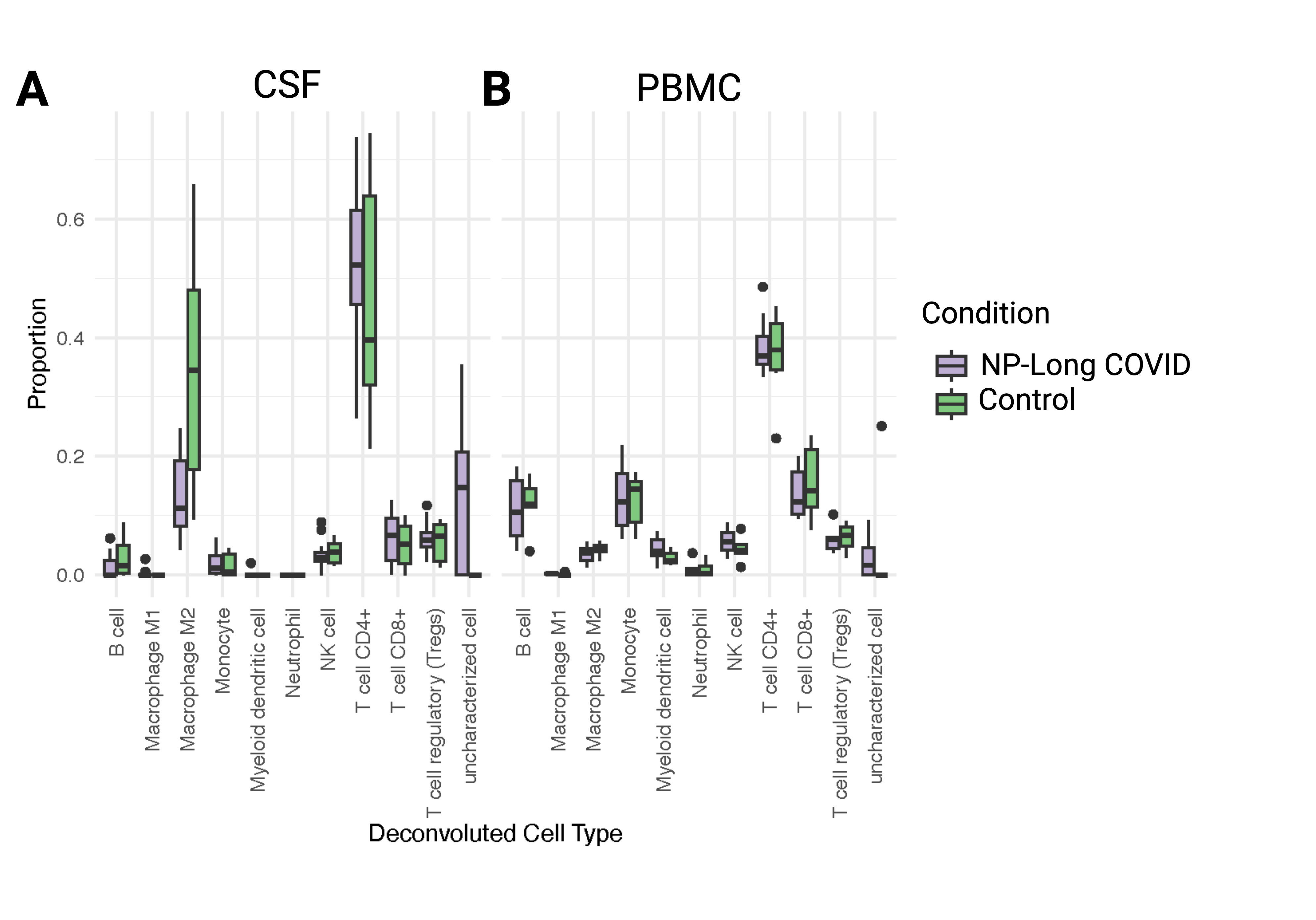

Supplement: jiaf468_Supplementary_Data [file jiaf468_supplementary_data.zip › 5 - Fig S1.png]
